# Looking for a proxy of the ionospheric turbulence with Swarm data

**DOI:** 10.1038/s41598-021-84985-1

**Published:** 2021-03-17

**Authors:** Paola De Michelis, Giuseppe Consolini, Alessio Pignalberi, Roberta Tozzi, Igino Coco, Fabio Giannattasio, Michael Pezzopane, Georgios Balasis

**Affiliations:** 1 Istituto Nazionale di Geofisica e Vulcanologia, 00143 Rome, Italy; 2 INAF-Istituto di Astrofisica e Planetologia Spaziali, 00133 Rome, Italy; 3 IAASARS-National Observatory of Athens, 15236 Athens, Greece

**Keywords:** Space physics, Astrophysical plasmas

## Abstract

The present work focuses on the analysis of the scaling features of electron density fluctuations in the mid- and high-latitude topside ionosphere under different conditions of geomagnetic activity. The aim is to understand whether it is possible to identify a proxy that may provide information on the properties of electron density fluctuations and on the possible physical mechanisms at their origin, as for instance, turbulence phenomena. So, we selected about 4 years (April 2014–February 2018) of 1 Hz electron density measurements recorded on-board ESA Swarm A satellite. Using the Auroral Electrojet (AE) index, we identified two different geomagnetic conditions: quiet (AE < 50 nT) and active (AE > 300 nT). For both datasets, we evaluated the first- and second-order scaling exponents and an intermittency coefficient associated with the electron density fluctuations. Then, the joint probability distribution between each of these quantities and the rate of change of electron density index was also evaluated. We identified two families of plasma density fluctuations characterized by different mean values of both the scaling exponents and the considered ionospheric index, suggesting that different mechanisms (instabilities/turbulent processes) can be responsible for the observed scaling features. Furthermore, a clear different localization of the two families in the magnetic latitude—magnetic local time plane is found and its dependence on geomagnetic activity levels is analyzed. These results may well have a bearing about the capability of recognizing the turbulent character of irregularities using a typical ionospheric plasma irregularity index as a proxy.

## Introduction

The ionospheric F-region can be rich in plasma density irregularities that cover a large range of scales, from a few meters to hundreds of kilometers (see, i.e.,Ref.^1^). They are generated mainly at the bottomside ionosphere but can extend vertically reaching altitudes of the order of 2000–3000 km^2^. The high-latitude ionosphere is one of those ionospheric regions where we can find intense plasma density irregularities caused by different processes mainly associated with auroral activities and plasma dynamics. This ionospheric region is indeed characterized by continuous interactions with diverse magnetospheric regions to which it is connected by magnetic field lines. Due to the direct connection between magnetosphere and ionosphere, the study of the origin and evolution of plasma irregularities can be quite complicated. Possible source mechanisms, which are typical of the high-latitude regions are for example, the variations of the electric current systems flowing both parallel and perpendicular to the Earth’s magnetic field^3^, the plasma convection^4^, the particle precipitation along the magnetic field lines^5,6^ and the thermospheric heating^4^. Each of these mechanisms is responsible for the generation of irregularities at different scales. For example, it has been suggested^5^ that the low-energy electron precipitation is one of the possible sources of irregularities at large-scale (> 10 km) but also processes like the $\vec {E} \times \vec {B}$ gradient drift instability and current convective instability can both directly and indirectly be responsible for plasma density and electric field irregularities at large scales. Conversely, strong field-aligned currents can lead to the formation of smaller plasma density irregularities, i.e., of the order of meters, which can be found mainly in the plane perpendicular to the magnetic field^3,7^. In the past, it has been suggested that irregularities can cascade from large scales to small scales due to the occurrence of turbulent phenomena^4^. In this case, power spectra are well described by power laws that extend over a wide range of scales: from kilometers down to a few meters. The slope of these spectra, the so-called *spectral index*, has been evaluated using different measurements covering various wavelength regimes. In the case of electron density data, recorded both on the ground and in space at different latitudes, values ranging between 1.5 and 2.5 have been obtained for the spectral index^1,8–11^. However, beyond the different spectral index values obtained over the years, the existence of a power law suggests scale invariance, meaning that the same physical process is responsible for the formation of irregularities at different scales^7^. Of course, the observed values of the spectral index may depend on different factors like, e.g., latitude or altitude. Furthermore, since different spectral indices may be proxies of several underlying physical processes, it is important to discuss them in connection with the selected ionospheric region.

In this work, we investigate the scaling features of the electron density fluctuations at high latitudes, in both hemispheres, recorded on-board one of the three ESA-Swarm satellites^12^ under two different geomagnetic activity conditions. This work is a generalization of a single case study recently published^13^ where, for the first time, it was suggested a possible link between the high values of Rate Of change of the electron Density Index (RODI) and some specific features of the electron density fluctuations for a period of 7 days around the 2015’s St. Patrick’s Day storm. In this study, we consider an extended dataset covering about 4 years (April 2014–February 2018), where we identify quiet and active geomagnetic conditions. The findings suggest the existence of two families of electron density irregularities characterized by different scaling properties and different values of RODI. The analysis focuses on the middle- and high-latitude regions and for the first time climatological maps of the probability densities of the two families in the magnetic latitude—magnetic local time plane are presented as a function of geomagnetic activity for both the hemispheres. Thus, our findings confirm what has been achieved in the case of a single storm and, at the same time, demonstrate that the existence of the two families is independent on the geomagnetic activity level. What changes is the weight of one family compared to the other as a function of the geomagnetic activity level and their probability density distributions in the magnetic latitude–magnetic local time plane. The results also confirm the possibility that RODI, which quantifies the relevance of ionospheric irregularities, may provide information on the features of the electron density fluctuations and on the possible physical mechanisms at their origin, as for instance, turbulence phenomena. This is of great importance in the framework of space weather. Indeed, ionospheric plasma density irregularities can strongly influence the quality of electromagnetic signals that propagate in space and consequently can influence the performance of those systems which are based on their propagation as for example the global navigation satellite systems (GNSS) and the global positioning system (GPS) on which many of our infrastructures depend either directly or indirectly^14^. The identification of regions characterized by turbulence processes could be the keystone for predicting where the degradation and loss of GPS signals have a higher probability to occur.

## Data

The present work focuses on the analysis of the scaling features of the electron density fluctuations in the mid- and high-latitude F-region ionosphere under two different conditions of geomagnetic activity. For this reason, we consider 1 Hz electron density measurements collected by the Langmuir Probes (LPs) on-board ESA Swarm A satellite, during a period of about 4 years (from April 2014 to February 2018). Using the Auroral Electrojet (AE) index, which is a good proxy of the geomagnetic activity level at mid/high latitudes, we select two distinct datasets corresponding respectively to geomagnetically quiet (AE < 50 nT) and active (AE > 300 nT) periods. The electron density measurements, retrieved from the ESA ftp repository (ftp://swarm-diss.eo.esa.int) are successively used to evaluate RODI, which is defined as the standard deviation of the Rate of change Of electron Density (ROD) in a sliding window of fixed size, in our case of 10 s^13,15,16^. We focus our attention on mid- and high-latitude ($>|50|^\circ$) regions in the Northern and Southern Hemisphere analyzing data in the Quasi-Dipole (QD) latitude^17^ and magnetic local time (MLT) coordinates.

## Method of analysis

One of the most striking features of turbulent signals is the scale-invariant nature of the fluctuations shown at different spatial and temporal scales. Indeed, turbulence is an inherently multiscale phenomenon, showing fluctuations either in space or in time (or both) and resulting in a noisy and chaotic dynamics. These multiscale fluctuations are due to the fact that in turbulent media the disturbances/excitations occur at macroscopic scales that are far from the microscopic scales at which dissipation takes place. According to the general picture of the Richardson’s cascade, turbulent media develop an inertial range where the mix of excited spatial and temporal scales is characterized by specific symmetry features (a phenomenon known as scale invariance). In that range, the energy injected at the largest scale is transferred to the smallest ones (where dissipation occurs) without a significant dissipation. The manifestation of scale invariance in a fluctuation field is the absence of any characteristic length or timescale for the emerging fluctuation structures, i.e., the occurrence of self-similarity in a statistical sense. In the case of a turbulent signal, a simple way to investigate the scaling features of the moments of the distribution of signal increments at different spatial/temporal scales is represented by the so-called $q^{th}$-order structure function at scale *l*, namely $S_q(l)$. Here, we compute the generalized $q^{\mathrm {th}}$-order structure function of the electron density $N_e$ as a function of the time delay $\tau$ , i.e.,

1 $$\begin{aligned} S_q(\tau ) = \langle \mid N_e(t+\tau )-N_e(t)\mid ^q\rangle , \end{aligned}$$

where *t* is the time and $\langle ...\rangle$ stands for a statistical average.

In presence of scale invariance $S_q(\tau )$ has a power-law behavior as a function of $\tau$:

2 $$\begin{aligned} S_q(\tau ) =\tau ^{\gamma (q)}, \end{aligned}$$

where $\gamma (q)$ is the so-called scaling exponent, which allows us to characterize the scaling nature of the increments of the signal under investigation (in our case $N_e$).

Here, we investigate the first- and second-order scaling exponents. The first-order scaling exponent $\gamma (1)$, also known as Hurst exponent (*H*)^18^, is a proxy of the persistent character of the analyzed time series increments, i.e., of the long-term memory of the time series^19^. It can assume any value in the range between 0 and 1. When the time series are characterized by values of *H* greater than 0.5, this means that the increments of the time series tend to cluster in a direction, and positive increments are more probably followed by positive increments while negative increments are more probably followed by negative ones. In this case the time series are characterized by a long-term positive autocorrelation. Conversely, when the scaling features of series are characterized by values of *H* lower than 0.5, the increments of the series tend to switch between positive and negative values, namely a positive increment is more likely to be followed by a negative one and vice-versa. In this case, the time series have the tendency to return to a long-term mean. Lastly, a value of the Hurst exponent equal to 0.5 indicates an absence of correlation in the increments (which are representative of fluctuations at the investigated time scale) of a time series that happens, for example, in the case of a Brownian time series.

The second-order scaling exponent, $\gamma (2)$, provides us information on the spectral features of the signal under investigation. In particular, for signals whose Fourier power spectral density (PSD) functions follow a power law behavior,

3 $$\begin{aligned} PSD(f) \sim f^{-\beta }, \end{aligned}$$

the power spectral density exponent, $\beta$, is related to the second-order structure function scaling exponent as follows

4 $$\begin{aligned} \beta = \gamma (2)+1. \end{aligned}$$

This is a consequence of the Wiener-Khinchin theorem relating the power spectral density of a signal to its autocorrelation function^20^. The study of the second-order scaling exponent is important for understanding the different physical processes (turbulence processes, stochastic processes, avalanche processes and so on) at the origin of a measured signal.

From a practical point of view, it is worth highlighting that the electron density time series recorded by Swarm satellite, like many real time series, are generally non-stationary, reflecting the diverse physical processes occurring in the different regions crossed by the satellite along its orbits. However, stationarity may occur locally, i.e., the scaling features may acquire a local character as in the case of a multifractional Brownian motion^21–23^. To evaluate the local scaling features of the electron density time series, we apply a local structure function method based on the detrended structure function analysis proposed by De Michelis et al.^24^. According to this method we use overlapping moving windows to remove possible large-scale variations in the time series, which can affect the correct estimation of the scaling features. In detail, we fix an overlapping moving window of *N* points and consider the portion of the time series falling in this window. We detrend this portion of the time series using a linear function and successively apply the classical structure function analysis. Here, we use a moving window of size equal to $N=301$ points that means a moving window of 301 s. Lastly, the same analysis is repeated on the new window obtained by shifting the previous window of 1 point, i.e., of 1 s.

Taking into account the size of the moving window, we can only evaluate the first two structure functions and consequently only the first two scaling exponents ($\gamma (1)\equiv H$ and $\gamma (2)$). Indeed, to obtain a good estimation of a scaling exponent the corresponding structure function must be computed on a window characterized by a size at least of the order of $10^q$. The structure functions are evaluated for different values of the time delay $\tau$ chosen in the range between 1 s and 40 s. After evaluating the first- and second-order structure functions in each moving window, we can estimate the first- and second-order scaling exponents whose values are associated with the position of the satellite in the instant of time corresponding to the center of the considered time window. The error associated with the first- and the second-order scaling exponents is approximately 7% and < 10%, respectively. These errors have been estimated via both a simulation of multifractional Brownian motions as described in Consolini et al.^23^ and a fitting procedure.

Concerning the stationarity of the analyzed time series, we can observe that the reduction of the time interval over which measurements are acquired corresponds to decrease the possibility of observing fast variations. Furthermore, since in several cases the spectral features associated with the actual measurements are characterized by power-law spectral densities with a spectral exponent $\beta$ in the range from − 3 to − 1, the corresponding time series are generally non-stationary with stationary increments in a range of time scales bounded below and above^25^. Thus, in our case the structure function analysis can be considered to be reliable. Moreover, although the analysis is done in the time domain, we can assume that it is also valid in the spatial one. Indeed, the analysis is applied to a range of time scales corresponding to those ranges where low-frequency temporal fluctuations are mainly due to Doppler-shifted and stationary spatial variations (see e.g., Refs.^26,27^ and references therein). This means that the scaling properties, that we find and that are valid in the range 1–40 s, can be considered valid also in the spatial domain which ranges between $\sim 8$ and $\sim 300$ km, considering the orbital velocity of Swarm satellite that is $\sim 7.6$ km/s.

Using the two first scaling exponents, *H* and $\gamma (2)$, it is possible to introduce an intermittency parameter, *I*, computed according to the following expression,

5 $$\begin{aligned} I=2H-\gamma (2). \end{aligned}$$

This parameter provides a measure of the inhomogeneity with which the energy is distributed in the cascade process from the largest to the smallest scales. More precisely, intermittency is a measure of the relevance of anomalous scaling features related to a multifractal character of the signal increments. It is a measure of the departure from a simple scaling, i.e.

6 $$\begin{aligned} S_q(\tau ) \ne \left( S_p(\tau )\right) ^{q/p}, \end{aligned}$$

being $\mathrm {d}^2\gamma (q)/\mathrm {d}q^2 \le 0$ $\forall$ moment orders $p\ \mathrm {and} \ q$. In other words, the scaling exponents do not follow a linear dependence on *q*, but show a convex trend. High values of intermittency in a time series are generally due to a non uniform distribution of the energy at all the time scales and manifest in temporal bursts. On the other hand, if the time series refers to a spatial crossing, the occurrence of intermittency can be related to a multifractal structure of the energy distribution in space as it occurs in the case of intermittency in turbulence.

## Results and discussion

To limit a considerable loss of points due to the moving window approach necessary to evaluate the first- and second-order scaling exponents, we evaluate them using a single electron density time series covering the whole selected period, 47 months with a time resolution of 1 s. In this way, the time series relative to the first- and second-order scaling exponents have only 300 points less than the original one. Indeed, due to the moving window applied to evaluate the scaling exponents, the scaling exponent time series begin and stop 150 points after and before the original electron density time series, respectively. Clearly, data refer to the entire globe and to different values of AE index. Thus, we select two different datasets, consisting of values of the scaling exponents associated with AE < 50 nT and with AE > 300 nT, respectively. Successively, we divide the QD-Latitude–MLT plane into a regular grid of $1^\circ \times 4$ min and compute polar view maps applying a weighted Gaussian interpolation scheme.

**Figure 1:**
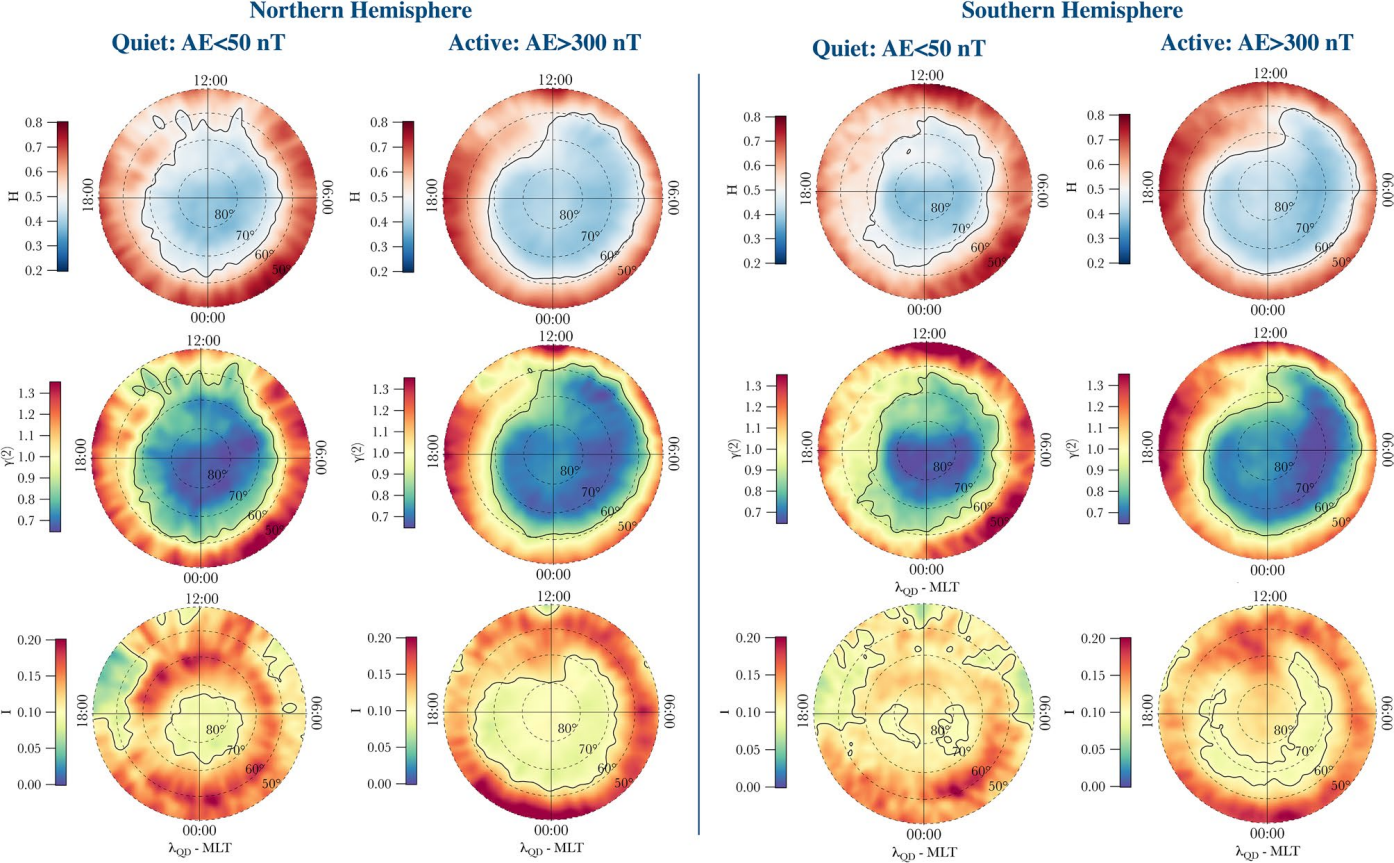
From top to bottom and from left to right: maps of the first two scaling exponents (*H*) and $\gamma (2)$ and intermittency (*I*) during quiet (AE < 50 nT) and active (AE > 300 nT) periods in the Northern and Southern Hemisphere. Maps are in the QD-latitude and magnetic local time (MLT) coordinate system. The concentric dashed circles represent contours of magnetic latitude, separated by $10^ \circ$. The black contours correspond to $H=0.5$, $\gamma (2)=0.9$ and $I=0.1$ in the maps of the first-, second-order scaling exponent and intermittency, respectively.

Figure 1 reports polar maps of the first-order scaling exponent at the top, the second-order scaling exponent in the middle and intermittency associated with the signal at the bottom. All maps are in QD-latitude and MLT coordinates, where noon is at the top and midnight at the bottom. They refer to the Northern (QD-latitude $> 50^\circ$) and Southern Hemisphere (QD-latitude $<-50^\circ$).

Polar maps of the first-order scaling exponent reveal the change of the scaling properties of $N_e$ fluctuations with latitude, MLT, and geomagnetic disturbance level. In particular, they show an antipersistent ($H<$ 0.5) character at high latitudes and a persistent one ($H>$ 0.5) at mid latitudes. This agrees with our earlier observations^13^ obtained analyzing the St. Patrick’s event only. Thus, the current climatological study confirms that $N_e$ fluctuations have an antipersistent character mainly inside the auroral oval and the polar cap where turbulent fluctuations can be generated by strong shear flows and gradient drifts due to plasma convection.

Polar maps of the second-order scaling exponent show a dependence on latitude, MLT, and slightly also on geomagnetic activity level. The values of this exponent range between 0.6 and 1.4 and the most interesting result is that the regions where $N_e$ fluctuations are antipersistent are mainly characterized by values of the second-order scaling exponent lower than 0.9 (black line). Considering the relationship between the second-order scaling exponent and the spectral exponent of the power spectral density (Eq. 4), this means that the $N_e$ fluctuations characterized by an antipersistent behavior are often associated with spectral exponents lower than 2. These spectral features of the electron density fluctuations agree with results by De Michelis et al.^13^. However, we can notice that the second-order scaling exponent is characterized by values which tend to be slightly different in the high-latitude regions. For example, in the polar cap region, the second-order scaling exponent is characterized by a value around 0.66, meaning electron density fluctuations whose power spectral density has a spectral exponent $\beta \simeq 5/3$. In the auroral regions, the second-order scaling exponents are, on the other hand, characterized by a spectral exponent $\beta \sim 1.9 - 2$. Conversely, the regions outside the auroral oval are characterized by $N_e$ fluctuations with a persistent character that are often associated with spectral exponents greater than 2. These features, which depend on the magnetic latitude and magnetic local time, are found during quiet and active periods and therefore seem to be a well-defined characteristic of $N_e$ fluctuations of the high latitude ionosphere.

Moving to the intermittency features, polar maps show that the region characterized by the highest values of intermittency tends to contract towards the pole or to expand towards the equator according to the level geomagnetic activity, i.e., moving from quiet to active periods. This suggests that the low-latitude edge of the auroral oval can be a region where turbulence processes may play a key role in the plasma dynamics and where the energy cascade is unevenly transferred from large to small scales. This assertion can be made on the basis of the relation existing between the spectral features of passive scalar quantities, as it is expected to be the electron density, and the ones corresponding to the velocity field in the case of turbulent media^28,29^. Thus, under the assumption of a definite link between the electron density fluctuations and the plasma velocity ones, we can observe that in these regions the energy dissipation associated with the turbulent fluctuations is strongly inhomogeneous being indeed localized in space.

**Figure 2:**
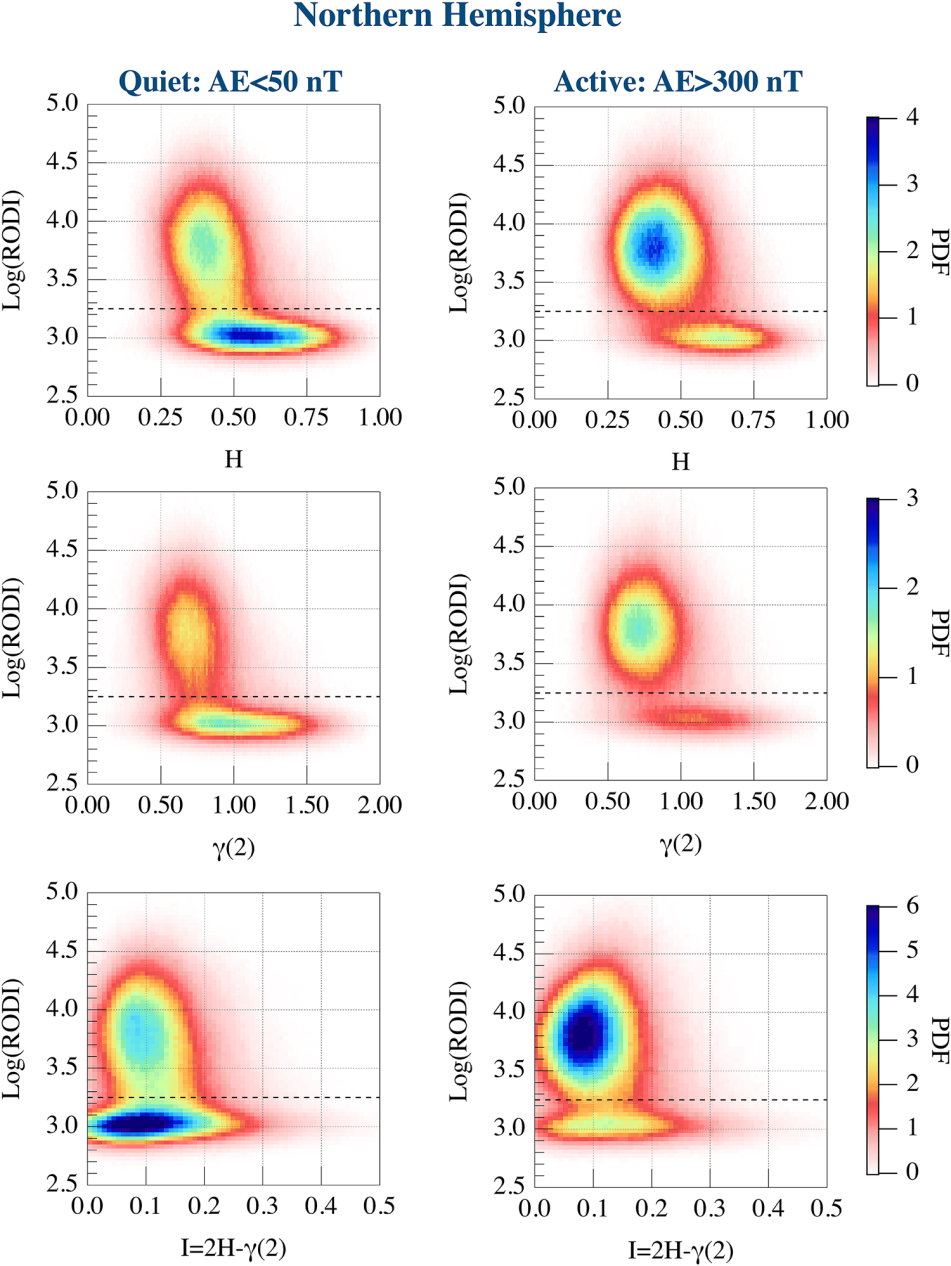
From top to bottom: joint probability distributions in the Northern Hemisphere between RODI and the first-order scaling exponent (first row), the second-order scaling exponent (second row) and intermittency (third row), respectively, obtained considering quiet and active periods. The black horizontal dashed lines shown in each panel correspond to $\mathrm {Log(RODI) }=3.25$.

**Figure 3:**
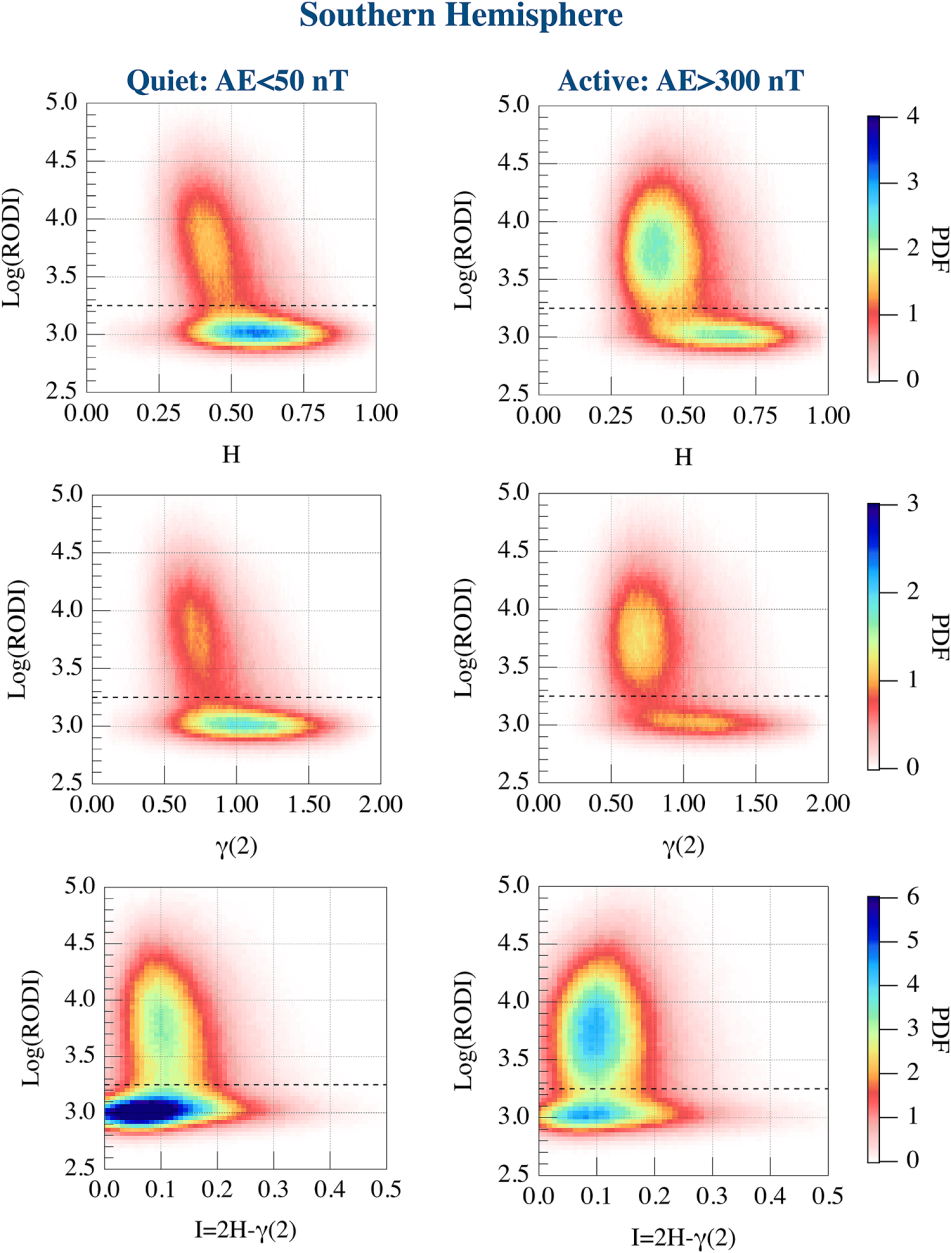
From top to bottom: the joint probability distributions in the Southern Hemisphere between RODI and the first-order scaling exponent (first row), the second-order scaling exponent (second row) and intermittency (third row), respectively obtained considering quiet and active periods. The black horizontal dashed lines shown in each panel correspond to $\mathrm {Log(RODI) }=3.25$.

Our previous analysis on the St. Patrick’s Day storm^13^ suggested a possible relationship between the high values of RODI and some specific features of $N_e$ fluctuations. To further investigate this aspect we repeat the analysis done by De Michelis et al.^13^ for each of the two different levels of geomagnetic activity in both the hemispheres. Specifically, we evaluate the joint probability distributions between RODI and the first-order scaling exponent, the second-order scaling exponent and intermittency, respectively. Corresponding results are reported in Figs. 2 and 3 in the case of quiet and active periods in the Northern and Southern Hemisphere, respectively. The study confirms from a statistical point of view that the joint probability distributions identify two families which are characterized by different scaling properties of $N_e$ fluctuations. High values of RODI are generally associated with $N_e$ fluctuations characterized by an antipersistent ($H<0.5$) behavior and a second-order scaling exponent $\gamma (2) < 1$. Conversely, low values of RODI are mainly associated with $N_e$ fluctuations characterized by a persistent ($H>0.5$) behavior and a second-order scaling exponent $\gamma (2) >1$. However, it should be noted that some of the low values of RODI can be also associated with antipersistent $N_e$ fluctuations. These two families can be identified in both hemispheres and their existence is independent on the geomagnetic activity level. To clarify the meaning of persistency in our context we can say that a persistent signal tends to generate large scale fluctuations (structures), while antipersistency has to be associated with the formation of small scale structures. Thus, the generation of irregularities at small scales should be associated with the antipersistent character of the analyzed signal ($H<0.5$). The value of RODI that reasonably seems to indicate the separation between the two families is the one for which $\mathrm {Log(RODI) }=(3.25 \pm 0.05)$. This threshold value is obtained by determining the average position of the minima between the power density functions (PDFs) of the two families along the y axis ($\mathrm {Log(RODI)}$) in the range between $3< \mathrm {Log(RODI)}<3.5$ and considering all the analyzed quantities and cases. It is highlighted by the horizontal black dashed lines in each panel of Figs. 2 and 3. This value is slightly different from what was previously achieved by analyzing the St. Patrick’s Day storm^13^ where we found a value around $\mathrm {Log(RODI) }=3.5$. The difference is due to a more rigorous methodology in determining the locus of minimal superposition between the two families that it has been possible thank to a wider statistics.

**Figure 4:**
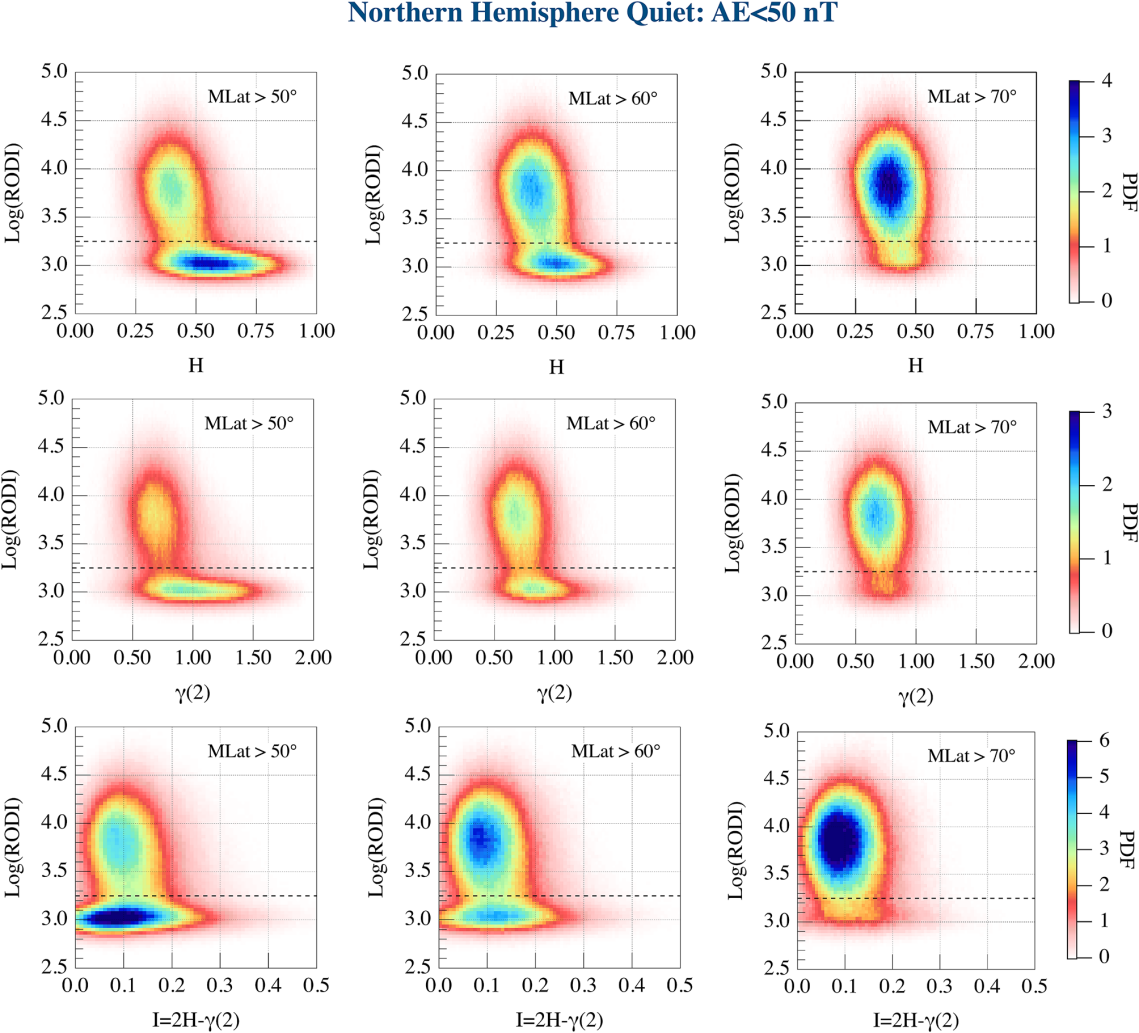
From top to bottom: the joint probability distributions in the Northern Hemisphere between RODI and the first-order scaling exponent (first row), the second-order scaling exponent (second row) and intermittency (third row), respectively obtained considering the quiet period, organized for different minimum QD-latitude values. The black horizontal dashed lines shown in each panel correspond to $\mathrm {Log(RODI) }=3.25$.

In order to establish whether the existence of the two families has any latitudinal dependence, we consider progressively smaller portions of the polar region. As an example, in the first column of Fig. 4 we report the joint probability distributions between RODI and the first-order scaling exponent (first row), the second-order scaling exponent (second row) and intermittency (third row), respectively obtained considering QD-latitudes higher than $50^\circ$ in the Northern Hemisphere during quiet period. The next 2 columns display the plots relative to the same joint probability distributions evaluated considering different values of the minimum latitude. What Fig. 4 points out is the existence of a dependence on latitude of the relative weight between the two different families. The family of electron density fluctuations with an antipersistent behavior and a second-order scaling exponent $\gamma (2)<1$ associated with high RODI values is more definite at high latitudes. The family of electron density fluctuations with a persistent behavior and a second-order scaling exponent $\gamma (2)>1$ associated with low RODI values disappears for QD-latitudes higher than $65^\circ{-}70^\circ$.

Thus, these figures suggest that high values of RODI are mainly associated with $N_e$ fluctuations characterized by scaling properties which recall those resulting from fluid-type turbulence processes. Conversely, other processes (a turbulence of different origin or other instability mechanisms) could be at the origin of the $N_e$ fluctuations associated with the other family. Moreover, both families seem to show a similar degree of intermittency which suggests that the associated fluctuation fields are non homogeneous. Similar results are obtained analyzing the disturbed period for the Northern Hemisphere and the quiet and disturbed periods for Southern Hemisphere.

**Figure 5:**
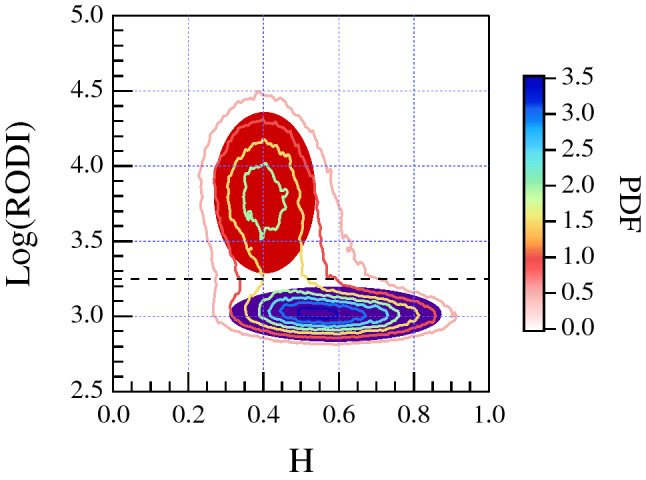
Selection of the two families, indicated in red and purple, in the case of the the joint probability distribution between RODI and the first-order scaling exponent (*H*) evaluated for magnetic latitudes higher than $50^\circ$ in the Northern Hemisphere (contour lines) for the quiet period.

**Figure 6:**
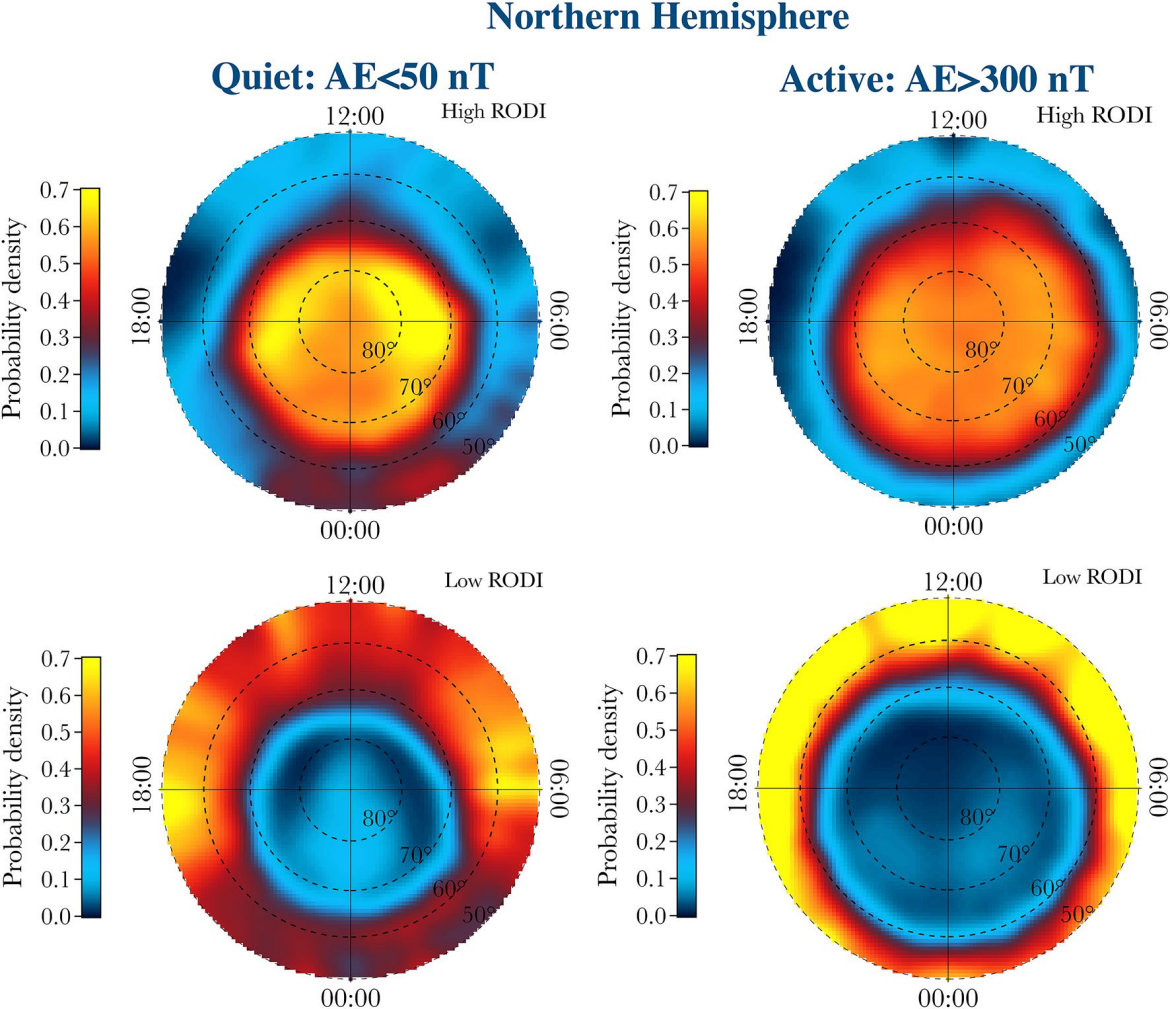
Distributions of the probability densities of the two families in the QDLat–MLT plane associated with high (top panels) and low (bottom panels) values of RODI in the Northern Hemisphere for quiet and active periods, respectively.

**Figure 7:**
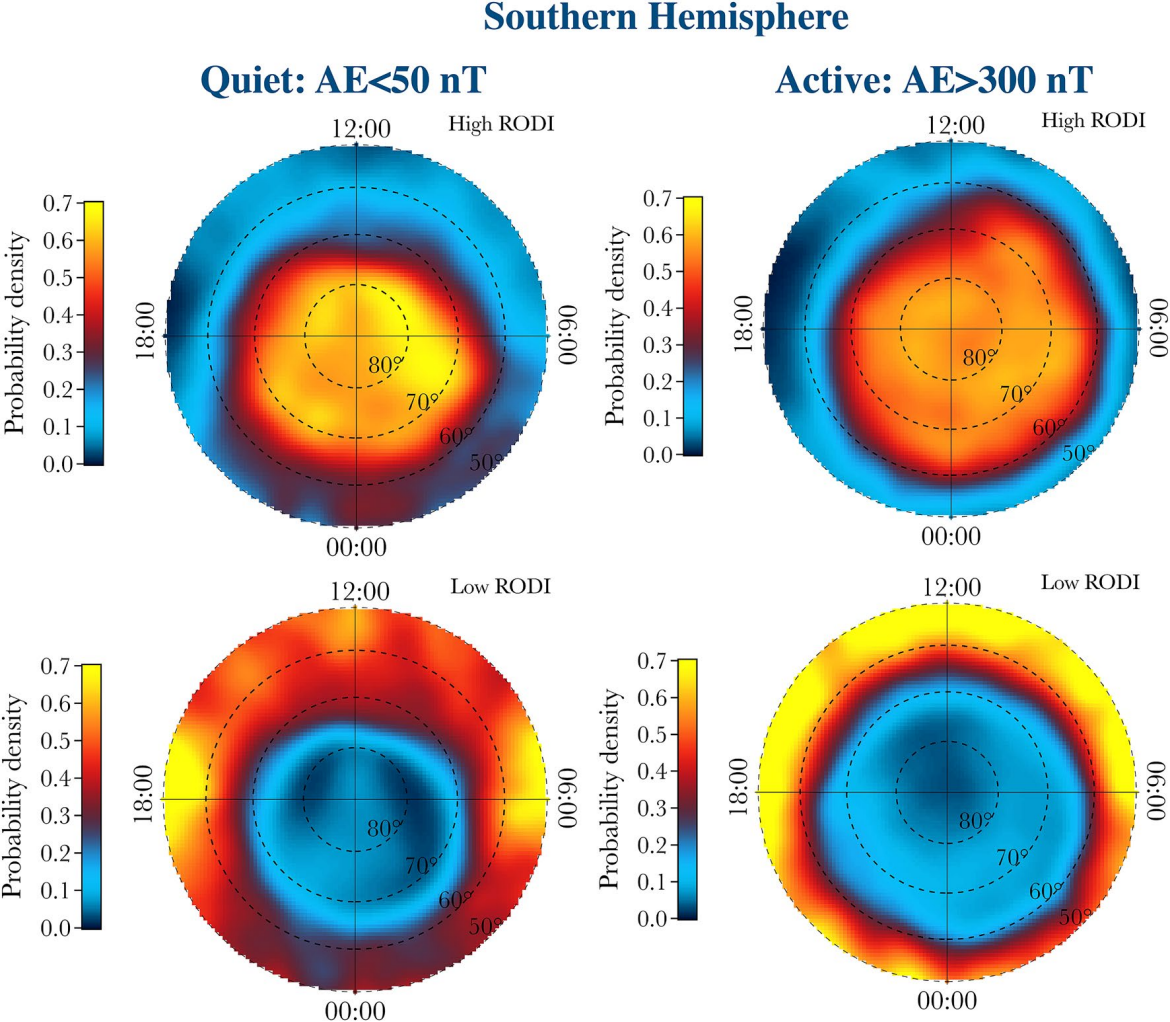
Distributions of the probability densities of the two families in the QDLat–MLT plane associated with high (top panels) and low (bottom panels) values of RODI in the Southern Hemisphere for quiet and active periods, respectively.

To visualize the distributions of these two different families in the QDLat–MLT plane we try to separate them. Figure 5 reports a sketch of the identification of the two families, indicated in red and purple, done using the joint probability distribution between RODI and the first-order scaling exponent (*H*) evaluated for magnetic latitudes higher than $50^\circ$ in the Northern Hemisphere for the quiet period. The selection between the two families is done using as a threshold for the RODI a value $\mathrm {Log(RODI)}=3.25$. The obtained spatial distributions of the probability densities of the two families in the QDLat–MLT plane associated with high (top panels) and low (bottom panels) values of RODI in the Northern Hemisphere during quiet and active geomagnetic periods are reported in Fig. 6. The same quantities relative to the Southern Hemisphere are reported in Fig. 7.

To compute the spatial distribution of the probability density function (PDF) reported in Figures 6 and 7 we use a kernel method based^30^ on a 2D Gaussian kernel, $k(u_x,u_y)$, defined as

7 $$\begin{aligned} k(u_x,u_y) = \frac{1}{2\pi \epsilon ^2}\exp \left[ -\frac{1}{2}\frac{u_x^2+u_y^2}{\epsilon ^2}\right] , \end{aligned}$$

being $\epsilon$ the kernel width (here of the order of 1 degree), while $u_x^2+u_y^2$ is the squared distance between the point where the probability is computed and the dataset points (i.e., $u_x^2+u_y^2= (x_i-x)^2+(y_i-y)^2$ where $\{(x_i, y_i)\}$ refers to the dataset). Thus, the PDF is given by

8 $$\begin{aligned} PDF(x,y) = \frac{1}{N}\sum _{i=1}^N k(x_i-x, y_i-y), \end{aligned}$$

where *N* is the number of points of the dataset. Furthermore, because the spatial distribution of the satellite measurements is not uniform in the QDLat–MLT plane, the obtained spatial distributions have been renormalized taking into account the corresponding ones of the satellite observations.

The location of these two different families in the QDLat–MLT plane clearly shows that the first population, which is characterized by antipersistency, $\beta < 2$ and high values of RODI, is mainly located at high latitude inside the auroral oval and the polar cap, while the second population, which is characterized by persistency, $\beta > 2$ and low values of RODI, is located at lower latitudes, mainly outside the auroral oval. With the increase of the geomagnetic activity, the distribution of the first population tends to expand towards lower latitudes, in perfect analogy with what happens to the auroral oval regions.

## Summary and conclusions

Plasma irregularities and turbulence processes characterize both the low- and high-latitude ionosphere. We used observations from Swarm constellation to study the scaling features of the electron density fluctuations in the mid- and high-latitude F-region ionosphere during geomagnetically quiet and active periods. The aim was to investigate the possibility that RODI, which quantifies the relevance of ionospheric irregularities, may provide information on the properties of the electron density fluctuations and on the possible physical mechanisms at their origin, such as, for instance, turbulence phenomena.

It is known that due to the solar wind—magnetosphere–ionosphere coupling, the high-latitude ionosphere is characterized by irregularities (plasma density fluctuations and structures) ranging from hundreds of kilometers down to meters. Processes such as for example plasma convection, particle precipitation, currents both parallel and perpendicular to the geomagnetic field, plasma instabilities and neutral fluid dynamics, such as gravity waves, have been proposed to account for the generation of these irregularities at large and intermediate scales, which mainly characterize the cusp region, the polar cap and the nightside sector auroral ionosphere. Under certain conditions these plasma irregularities can become unstable, producing scale-invariant structures via turbulent phenomena. Indeed, phenomena such as the $\vec {E} \times \vec {B}$ gradient drift instability (GDI) or the current convective instability (CCI), can both directly and indirectly produce a cascade process through which smaller size irregularities are generated^7,31^. These scale-invariant features of plasma density fluctuations associated with irregularities manifest in different spectral features i.e., in different power spectral exponents, and persistency features.

One of the main findings of our statistical analysis is that we can clearly identify two different families of plasma density fluctuations characterized by different mean values of scaling exponents and RODI. This finding suggests that, in general, two main different classes of physical phenomena can be at the origin of the different scaling features. Furthermore, a clear different localization of the two families in the QDLat–MLT plane is found. The first population, which is characterized by antipersistency, $\beta = \gamma (2)+1< 2$ and high values of RODI, is mainly located inside the auroral oval where particle precipitation dominates. The second population, which is characterized by persistency, $\beta > 2$ and low values of RODI, is located at low latitude, mainly outside the auroral oval. These findings suggest that, in general, the first population could be due to turbulent mechanisms generated by GDI. Indeed, the observed average value of the spectral density exponent $\beta \sim 1.7$ is in agreement with previous findings^2,9,32^ and with theoretical predictions for this turbulent mechanism^7^. Furthermore, the polar cap and, in particular, its trailing side are regions where due to the large-scale convection GDI may be an active phenomenon. At the same time, we cannot exclude that other mechanisms could be at the origin of the observed spectral features, as for instance the occurrence of coherent structures and nonlinear wave interactions^32^. However, we remark that coherent structures are also a product of an intermittent turbulent cascade that is a common feature of strong turbulence, so that the observed spectral features could be due to the occurrence of an intermittent strong turbulence generated by GDI.

For what concerns the second population, this is characterized by spectral features ($\beta \sim 2$) that remind random turbulent fluctuations in regions where a density enhancement is observed in correspondence with plasma coming from the inner magnetospheric regions (see, e.g.^32,33^). The intermittency degree associated with this population seems to be slightly lower than that of the first one, suggesting that the observed density fluctuations could be due to mechanisms different from strong/fully developed turbulence. To support this hypothesis we remind that electron density could be considered as a passive scalar in turbulent medium. Indeed, it has been reported in the literature^34^ that passive scalar quantities can show a certain degree of intermittency also in the case of purely stochastic velocity fields, so we can speculate that the low degree of intermittency of this population with respect to the first one could be the counterpart of a more stochastic nature of the driving field, i.e., the electrostatic field^7^. However, we cannot exclude that other mechanisms such as CCI and Kelvin-Helmoltz instability could be responsible for the observed features. Thus, a careful discussion of this second population needs further theoretical/numerical investigations, including also the possible effects of neutrals^35^. Anyway, a detailed discussion of the different instability mechanisms at the origin of the observed scaling features requires the investigation of other physical quantities, such as magnetic and electric field fluctuations. Some preliminary analyses of the magnetic field scaling features as a function of different interplanetary magnetic field orientations^36^ and geomagnetic activity levels^24,37^ have shown that there is not a simple relationship between the scaling properties of these two physical quantities, suggesting that a more detailed analysis should be performed in order to identify and characterize the instability mechanisms at work. This is postponed to future works, being the aim of the present study limited to the analysis of the link between RODI and the electron density scaling features.

An implication of our analysis is the possibility to consider RODI as a proxy to identify different families of irregularities and the different nature of physical processes at the base of their origin. Specifically, our study showed that RODI could be used to detect plasma density irregularities caused by fully developed turbulent phenomena.

This is a positive outcome for space weather purposes. Indeed, ionospheric plasma density irregularities can strongly influence the quality of propagating electromagnetic signals in terms of delay, distortion and signal loss, adversely affecting systems as GNSS and GPS on which many of our infrastructures depend either directly or indirectly. Recently, it has been found^38^ that at high latitudes plasma density irregularities, and in particular those associated with large plasma gradients, play an important role in the GPS signal loss. In fact, the GPS signal loss events seem to occur with high probability when the electromagnetic signals propagate inside regions characterized by large electron density gradients. The climatological maps of GPS signal loss based on Swarm measurements reveal that they occur in the Northern Hemisphere mainly around the cusp region and along nightside auroral latitudes^38^, that are precisely the regions identified by our analysis in terms of families of electron density fluctuations characterized by antipersistency, a second-order scaling exponent $\gamma (2) <1$ and high values of RODI. This suggests that GPS signal loss events could be linked to the occurrence of turbulence processes characterizing the plasma density fluctuations and structures. The identification of regions characterized by turbulence processes could be the keystone for predicting the ones where the degradation and loss of GPS signals have a higher probability to happen.

## Data Availability

Swarm data can be accessed online (http://earth.esa.int/swarm). OMNI data can be accessed online (https://cdaweb.gsfc.nasa.gov/index.html/)
